# Natural Marine and Synthetic Xenobiotics Get on Nematode’s Nerves: Neuro-Stimulating and Neurotoxic Findings in *Caenorhabditis elegans*

**DOI:** 10.3390/md13052785

**Published:** 2015-05-06

**Authors:** Thora Lieke, Christian E. W. Steinberg, Jingjuan Ju, Nadine Saul

**Affiliations:** 1 Department of Biology, Freshwater and Stress Ecology, Humboldt-Universität zu Berlin, Späthstr. 80/81, 12437 Berlin, Germany; E-Mails: christian_ew_steinberg@web.de (C.E.W.S.); jjj0810@wmu.edu.cn (J.J.); nadines1976@aol.com (N.S.); 2 Key Laboratory of Environmental Medicine Engineering, Ministry of Education, School of Public Health, Southeast University, Nanjing 210009, China

**Keywords:** tetrabromobisphenol-A, dibromoacetic acid, acute environmental toxicology, neurobehavior, gene expression, GFP, ontogeny, *C. elegans*

## Abstract

Marine algae release a plethora of organic halogenated compounds, many of them with unknown ecological impact if environmentally realistic concentrations are applied. One major compound is dibromoacetic acid (DBAA) which was tested for neurotoxicity in the invertebrate model organism *Caenorhabditis elegans* (*C. elegans*). This natural compound was compared with the widespread synthetic xenobiotic tetrabromobisphenol-A (TBBP-A) found in marine sediments and mussels. We found a neuro-stimulating effect for DBAA; this is contradictory to existing toxicological reports of mammals that applied comparatively high dosages. For TBBP-A, we found a hormetic concentration-effect relationship. As chemicals rarely occur isolated in the environment, a combination of both organobromines was also examined. Surprisingly, the presence of DBAA increased the toxicity of TBBP-A. Our results demonstrated that organohalogens have the potential to affect single organisms especially by altering the neurological processes, even with promoting effects on exposed organisms.

## 1. Introduction

Xenobiotics of both, natural and synthetic sources can influence organisms in many different ways. Since more and more chemicals are released into the environment toxicological investigations about how these xenobiotics act on organisms become increasingly important. Up to 360 µg/g dry weights of organic halogens have been found in Swedish forests [1] and up to 35 µg/L of adsorbable organic bromine in Berlin lakes [2]. The amount found in industrial waste water has increased almost by factor 100 [3]. These figures clearly show the increasingly importance of realistic ecotoxicological investigations on how these xenobiotics act on organisms. In many past corresponding investigations, two important issues were often not well considered: on the one hand unrealistically high concentrations were usually used while screening for the toxic potential, on the other hand most tests were restricted to determining the LD_50_ and changes in lifespan. Only a few analyses have been conducted on neurological effects, although the nervous system is one of the primary targets for environmental chemicals and pollutions [4]. This aspect is gaining increasing significance, since many invertebrates lack real livers and even well-known hepatotoxins display major adverse neurotoxic actions, as recently exemplified with microcystin-LR in *Caenorhabditis elegans* (*C. elegans*) [5]. One of the main reasons, why neurological examination is often omitted, is that standardized neurotoxicity screening methods devolved by the WHO and US EPA use vertebrates like rats or mice. Thus, these tests are expensive, time-consuming, complex and not least ethically questionable. We have recently developed a protocol which allows the transfer of neurotoxicity screening methods to the invertebrate model organism *C. elegans* [6]. This allows integration of neurological content issues to toxicological screenings. Recently, we investigated the impact of two organohalogens, namely 2,2′,6,6′-tetrabromo-4,4′-isopropylidendiphenol (tetrabromobisphenol-A, TBBP-A) and dibromoacetic acid (DBAA), on the lifespan, growth and offspring of *C. elegans* in natural occurring concentrations [7]. The synthetic xenobiotic, TBBP-A, is used as flame retardant and was found in marine sediments and muscles [8]. It acts as an endocrine disruptor [9]; in particular, due to its stuctural similarity to thyroxin, also as immunosuppressive [10,11]. Changes in neuronal behavior in mice have also been reported by Nakajima *et al.* [12]. With *C. elegans*, TBBP-A acted in a hormetic way as low concentrations extended lifespan while higher concentration decreased it [7].

Dibromoacetic acid has both sources, artificial and natural ones. It is produced by marine algae, such as *Asparagopsis* sp. [13], but is also a by-product of freshwater disinfection [14,15]. It is a toxic, carcinogen and also neurotoxic character has been shown [16,17]. More recently, DBAA was found in considerable concentrations in freshwater, for instance in the Berlin waterways [2]. Therefore, it was of particular interest to identify a toxic potential. Interestingly, Saul *et al.* [7] demonstrated a stimulating effect of environmental realistic concentrations of DBAA to the lifespan of *C. elegans*. This underlines the importance to refer to environmental realistic concentrations when evaluating the interaction of chemicals with organisms. In this study, we found a stimulating effect for 0.1 µM TBBP-A and 50 µM DBAA, while 50 µM TBBP-A had a toxic effect. Furthermore, a microarray analysis revealed that both organobromines modulated different signaling and neurological processes. The aim of this study was to determine if changes in lifespan and growth are caused by differences in neuronal behavior and if microarray findings are also reflected in phenotypic life traits. Therefore, we analyzed the impact of the same concentrations and mixtures of TBBP-A and DBAA on the autonomic (locomotion, pumping, and defecation) and sensory (mechanical, chemical, and thermal) neurological behavior and on the molecular level via qRT-PCR. We hypothesized that neurological modulations are the underlying mechanisms for the observed stimulating or toxic effects of these organobromines, rather than modulations of biochemical variables such as enzyme or transporter activities or mobilization energy depots.

## 2. Results

### 2.1. Acute Exposure

Exposure of *C. elegans* to both chemicals alone and in mixture changes several behavioral traits as well as gene transcription.

#### 2.1.1. Locomotive Behavior

**Figure 1:**
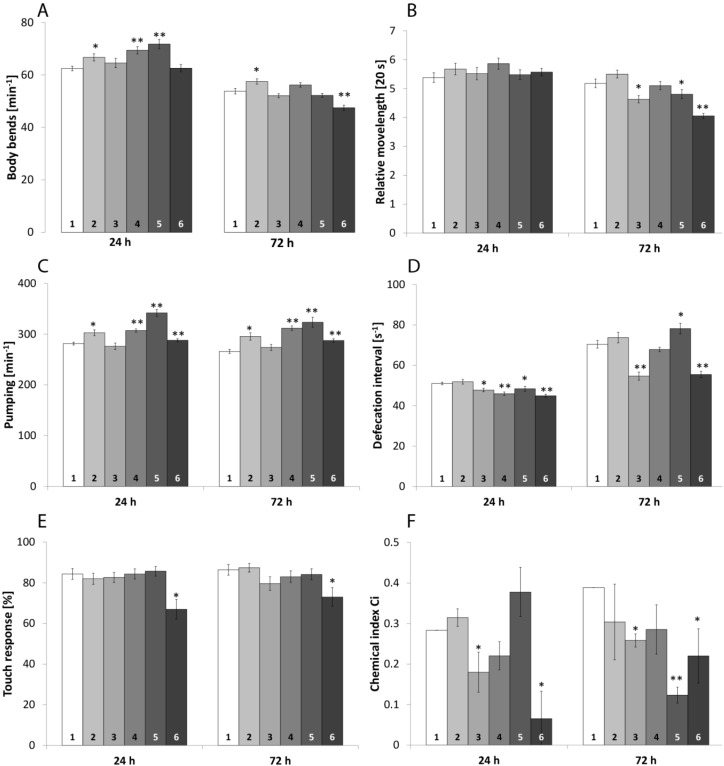
Neurophysiological behavior. The graphs show locomotive behavior (body bends (**A**) and relative movelength (**B**)); pumping frequency (**C**); defecation interval (**D**); mechanical sensory (**E**) and chemical index (**F**). **1**: Control, **2**: 0.1 µM TBBP-A, **3**: 50 µM TBBP-A, **4**: 50 µM DBAA, **5**: 0.1 µM TBBP-A + 50 µM DBAA, **6**: 50 µM TBBP-A + 50 µM DBAA. Significant changes to the control are given by * (*p* < 0.05) and ** (*p* < 0.001). Bars represent mean values ± SEM (One Way ANOVA (Holm-Sidak-method)).

The ability to move without stint is not only necessary to reach food but also to avoid predators and unfavorable conditions. Therefore, the impact of the chemicals on locomotive behavior was assayed using two aspects: on the one hand the number of body bends per minute, which reflects the capability to react to immediate stressors, such as mechanical tension. On the other hand, the relative movement speed was measured, reflecting the ability to reach food and escape predators or chemicals. Both aspects can be influenced in different ways, as shown for *ggb-1* mutants [18]. Untreated worms could perform about 62 bends per minutes after 24 h (Figure 1A) and the worms moved about five times their body length in 20 s (Figure 1B). After 72 h the speed of movement remained identical, but the body bend frequency decreased to 54 bends per minute. Incubation of 24 h with 0.1 µM TBBP-A, 50 µM DBAA and the mixture of both caused an increased body bend frequency. The mixture significantly increased the body bend frequency in relation to 0.1 µM TBBP-A. After 72 h, 0.1 µM TBBP-A still caused an increase, whereas the mixture of both high concentrated organobromines significantly decreased the frequency in relation to the control and to 50 µM DBAA. After 72 h incubation, both mixtures decreased the frequency significantly in relation to the single compounds.

The movement speed was significantly decreased by 50 µM TBBP-A and by both mixtures after 72 h. In addition, the mixture of 0.1 µM TBBP-A and DBAA significantly decreased the speed in relation to 0.1 µM TBBP-A alone and the mixture of both high concentrations reduced the speed in relations to both individual components. For detailed values, please refer to Table S1 and significances between mixtures and components are listed in Table 1.

#### 2.1.2. Pharynx Pumping

Food ingestion and thereby energy-uptake is essential for every animal and impairment can decrease survival and fitness. *C. elegans* feeds through a cycle of contraction and relaxation of the pharyngeal muscles, which can be monitored by up and down movement of the grinder. A decreased frequency reflects a smaller amount of energy that is available for maintenance and growth.

The pumping frequency of the pharynx of unimpaired nematodes was about 281 and 266 pumps per minute, after 24 h and 72 h, respectively (Figure 1C). Incubation with all substances except 50 µM TBBP-A led to a significant increased pumping frequency after 24 h and 72 h. Mixture of 0.1 µM TBBP-A and DBAA even significantly increased the frequency in relation to the individual components (Table 1), with exception of 50 µM DBAA after 72 h, while the second mixture increased the frequency referred to 50 µM TBBP-A but decreased it as compared to DBAA. Table S2 displays mean values, SEM and significances.

**Table 1:** Significances of mixtures in relation to the single compounds.

Neurophysiological Behavior	*t*_ex_	0.1 T + 50 D Relative to		50 T + 50 D Relative to	
		0.1 T	50 D	50 T	50 D
**Body bends**	24 h	*	-	-	*
	72 h	**	*	**	**
**Relative movelength**	24 h	-	-	-	-
	72 h	*	-	**	**
**Pumping**	24 h	*	**	*	*
	72 h	*	-	-	**
**Defecation**	24 h	*	-	*	-
	72 h	-	*	-	**
**Touch response**	24 h	-	-	*	*
	72 h	-	-	-	-
**Chemical index**	24 h	-	-	-	-
	72 h	-	*	-	-

***t*_ex_**: exposure time; **0.1 T**: 0.1 µM TBBP-A; **50 T**: 50 µM TBBP-A; **50 D**: 50 µM DBAA; **red**: significantly higher than the single substance; **green**: significantly lower than the single substance; Significant changes are given by * (*p* < 0.05) and ** (*p* < 0.001).

#### 2.1.3. Defecation

The more time ingested food remains in the intestine, the better the nutrients can be absorbed. Increased defecation reflects a diminished amount of energy that the nematodes can extract from the food and that is available for maintenance. The defecation cycle of *C. elegans* consists of three consecutive muscle contractions [19] of which the expulsion step can be monitored by using a stereomicroscope. The time between two cycles was measured.

As shown in Figure 1D, the period between two defecations was about 51 s after 24 h and about 70 s after 72 h for nematodes treated with dimethyl sulfoxide (DMSO). After 24 h, all substances except for 0.1 µM TBBP-A provoked a significant deceleration. The mixtures were also down regulated in reference to at least one of their components (Table 1). After 72 h exposure to 50 µM TBBP-A and the mixture of the high concentrated organobromines it caused a down-regulation while the other mixture caused an up-regulation. For details on mean value and SEM, please refer to Table S3.

#### 2.1.4. Mechanical Sensory Perception

Mechanical perception allows the nematode to interact with its environment and react to predators as well as food sources. Decreased perception therefore may lead to decreased ingestion and reduced energy. Mechanical sensory was monitored by touching the anterior end of the nematode with a fine hair.

Untreated nematodes showed a positive reaction to about 84% and 86% to nose touching (Figure 1E and Table S4). The nose-touch avoidance behavior was significantly affected by exposure to the mixture of 50 µM TBBP-A and DBAA. Both, after 24 h and 72 h, the percentage of positive reactions was drastically reduced in relation to control and likewise to the individual compounds (Table 1).

#### 2.1.5. Chemical and Thermal Sensory Perception

*C. elegans* detects and evaluates food sources as well as harmful substances through chemical perception. A reduced perception therefore not only decreases the amount of energy gained by ingestion but also increases the risk to contact chemical stressors. When the nematodes are grown on agar containing NaCl and were fed *ad libitum* during that period, they associate NaCl with food sources. For this, the fraction of animals that crawled towards the NaCl source was determined and the chemical index was calculated.

Untreated *C. elegans* had a chemical index of about 0.28 after 24 h of incubation and about 0.39 after 72 h. As shown in Figure 1F the ability to perceive NaCl dramatically decreased during every experimental design containing 50 µM TBBP-A, however, no statistical difference could be found between the mixture and its individual components (Table 1). In addition, the chemical index also decreased after 72 h of incubation with the mixture of 0.1 µM TBBP-A and DBAA. Detailed information on mean value, standard error of the mean, and significances are shown in Table S5. Thermotaxis behavior was assayed as well, however, due to high deviation, no significant changes were found. For details, please refer to the Supplements “Thermotaxis”.

#### 2.1.6. Neuromolecular Experiments

We hypothesized that transcriptional changes caused by the organobromines are the underlying mechanisms for the observed modulated neuronal behavior, rather than biochemical modulations. Therefore, we chose 17 genes, either representing different neuronal pathways or because they showed transcriptional changes in the previous performed microarray [7] and are involved in the examined behavior. For detailed information on description and function of the genes, please refer to Table 2.

**Table 2:** Description and function of examined genes.

Gene	Function
*ace-1* (abnormal AcetylCholinEsterase)	Acetylcholinesterase
*casy-1* (CAlSYntenin/Alcadein homolog)	Learning
*che-12* (abnormal CHEmotaxis)	Chemorezeptor
*dat-1* (DopAmine Transporter)	Dopamine-transporter
*dop-1* (DOPamine receptor)	D1-dopamine-receptor
*dop-3* (DOPamine receptor)	D2- dopamine-receptor
*eat-4* (EATing: abnormal pharyngeal pumping)	Vesicular glutamate transporter
*gbb-1* (GABAB receptor subunit)	GABA-receptor
*grd-12* (GRounDhog (hedgehog-like family))	Growth and movement
*hda-4* (Histone DeAcetylase)	Histone deacetylase
*hen-1* (HEsitatioN behavior)	Integration of sensory stimuli
*mec-10* (MEChanosensory abnormality)	Mechanical stimulation
*srab-6* (Serpentine Receptor, class AB (class A-like))	Needed for chemotaxis
*tph-1* (TryPtophan Hydroxylase)	Serotonin synthesis
*ttx-3* (abnormal ThermoTaXis)	For AIY interneurons
*unc-17* (UNCoordinated)	Vesicular acetylcholine transporter
*unc-47* (UNCoordinated)	Vesicular GABA transporter

Transcription of *ace-1* tended to increase after exposure to the high concentrations of both organobromines and their mixture, however, no statistical difference to the control could be determined. TBBP-A (0.1 µM) significantly decreased the transcription of *casy-1*, *gbb-1*, *grd-12*, *ttx-3* and *unc-47*, but increased the transcription of *unc-17* and *hen-1*. The high concentration of TBBP-A increased the synthesis of several mRNA, except from *eat-4*, were the amount was significantly decreased.

DBAA had a similar effect as 0.1 µM TBBP-A and led to a reduced gene-transcription in many cases. Only *hen-1*, *ace-1* and *srab-6* were induced, but in the case of *ace-1* no significant increase could be detected. Both mixtures induced numerous gene transcriptions. Nevertheless, *eat-4* and *grd-12* were repressed by the mixture containing 0.1 µM TBBP-A and *hen-1* and *mec-10* by the mixture containing 50 µM TBBP-A. For detailed information about the changes in transcription please refer to Table 3.

With exception of *hen-1*, *srab-6* and *unc-17*, the mixture containing 0.1 µM TBBP-A significantly increased the gene-transcription relative to both single compounds. For 50 µM TBBP-A and DBAA in the mixture a statistical difference to one of the single compounds was observed for nine genes. A statistical difference to both components was never detected. Significances are shown in Table 4.

**Table 3:** Relative fold changes (*f*) of gene transcriptions in relation to control.

Gene	0.1 µM TBBP-A	50 µM TBBP-A	50 µM DBAA	0.1 µM TBBP-A + 50 µM DBAA	50 µM TBBP-A + 50 µM DBAA
	mean ± SEM	mean ± SEM	mean ± SEM	mean ± SEM	mean ± SEM
***ace-1***	1.52 ± 0.66	4.02 ± 1.67	2.97 ± 1.34	1.28 ± 0.82	4.02 ± 2.21
***casy-1***	0.41 ** ± 0.13	1.75 * ± 0.32	0.73 ± 0.23	4.22 * ± 1.24	2.14 * ± 0.35
***che-12***	0.82 ± 0.23	1.33 ± 0.22	0.49 * ± 0.12	2.12 ** ± 0.21	0.99 ± 0.21
***dat-1***	0.59 ± 0.20	2.37 ± 0.73	1.20 ± 0.22	2.32 * ± 0.31	2.09 ** ± 0.14
***dop-1***	1.31 ± 0.46	3.06 ± 1.02	0.56 * ± 0.23	1.08 ± 0.33	1.11 ± 0.24
***dop-3***	1.09 ± 0.24	2.70 * ± 0.73	0.49 * ± 0.18	2.09 ** ± 0.17	1.62 * ± 0.20
***eat-4***	0.86 ± 0.29	0.64 * ± 0.18	0.40 ** ± 0.08	0.63 * ± 0.13	4.51 * ± 1.42
***gbb-1***	0.61 * ± 0.12	1.27 ± 0.21	0.49 * ± 0.11	1.48 * ± 0.22	1.20 ± 0.16
***grd-12***	0.47 * ± 0.16	0.75 ± 0.20	0.65 * ± 0.15	0.68 ** ± 0.07	0.70 ± 0.34
***hda-4***	1.15 ± 0.13	1.14 * ± 0.07	1.23 ± 0.15	1.53 * ± 0.19	1.26* ± 0.09
***hen-1***	2.01 ± 0.69	2.56 * ± 0.57	3.59 * ± 1.20	3.02 * ± 0.89	0.50 * ± 0.13
***mec-10***	0.95 ± 0.09	1.25 * ± 0.07	1.05 ± 0.10	2.29 ** ± 0.14	0.86 * ± 0.07
***srab-6***	0.78 *± 0.08	1.07 ± 0.11	1.83 * ± 0.28	2.17 ** ± 0.24	1.88 * ± 0.23
***tph-1***	0.85 ± 0.10	1.08 ± 0.07	0.80 ± 0.17	1.74 ** ± 0.14	1.05 ± 0.15
***ttx-3***	0.59 * ± 0.15	1.48 ± 0.33	1.43 ± 0.28	1.17 ± 0.17	2.26 * ± 0.56
***unc-17***	2.52 * ± 0.73	1.51 * ± 0.23	1.03 ± 0.10	2.37 * ± 0.34	1.68 * ± 0.25
***unc-47***	0.47 ** ± 0.06	3.76 * ± 1.01	0.75 * ± 0.12	5.85 * ± 2.02	2.53 ** ± 0.21

**Yellow**: 2.0 > *f* > 1.5; **red**: *f* >2.0; **light green**: 0.5 < *f* < 0.67; **green**: *f* < 0.5; significances are marked with * (*p* < 0.05) and ** (*p* < 0.001).

**Table 4:** Significances (*p*) for the relative fold changes of gene transcription of the mixtures in relation to the single compounds.

Gene	0.1 μM TBBP-A + 50 μM DBAA Relative to		50 μM TBBP-A + 50 μM DBAA Relative to	
	0.1 μM TBBP-A	50 μM DBAA	50 μM TBBP-A	50 μM DBAA
***ace-1***	n.s.	n.s.	n.s.	n.s.
***casy-1***	*	*	n.s.	*
***che-12***	*	**	n.s.	n.s.
***dat-1***	*	*	n.s.	*
***dop-1***	n.s.	n.s.	n.s.	n.s.
***dop-3***	*	**	n.s.	*
***eat-4***	n.s.	n.s.	n.s.	*
***gbb-1***	*	*	n.s.	*
***grd-12***	n.s.	n.s.	n.s.	n.s.
***hda-4***	n.s.	n.s.	n.s.	n.s.
***hen-1***	n.s.	*	*	n.s.
***mec-10***	**	**	*	n.s.
***srab-6***	*	n.s.	*	n.s.
***tph-1***	*	*	n.s.	n.s.
***ttx-3***	n.s.	n.s.	n.s.	n.s.
***unc-17***	n.s.	*	n.s.	n.s.
***unc-47***	n.s.	n.s.	n.s.	**

**n.s.**—not significant; * *p* < 0.05; ** *p* < 0.001; **red**: up-regulation; **green**: down-regulation.

#### 2.1.7. Fluorescence-Microscopic Observations of GFP Fusion-Proteins

Transcriptional changes do not necessarily mean changes in translation and protein amount, as many post-translational and post-transcriptional processes, such as mRNA and protein stability or degradation can influence the amount of protein. Therefore we used five *C. elegans* strains, where neuron-specific proteins were tagged with GFP, to evaluate the impact of the substances to protein synthesis as well as to the neurons: Dopaminergic neurons (BZ555 (*dat-1*:GFP)), glutamatergic neurons (DA1240 (*eat-4*:GFP)), GABAergic neurons (EG1285 (*unc-47*:GFP)), serotonergic neurons (GR1366 (*tph-1*:GFP)) and cholinergic neurons (LX929 (*unc-17*:GFP)). The fluorescence intensity of the whole nematode was measured using Image J software and the corrected total cell fluorescence (CTCF) was calculated. For more details on the strains please refer to Section 4.1 and for detailed information on the tagged genes to Table 2 and Table S6. In order to evaluate the impact of the substances to survival of the neurons, the number of neurons has been counted out. This was possible only for BZ555 and EG1285, as EAT-4 (DA1240) and TPH-1 (GR1366) are not expressed in each of the eight serotonin or glutamate neurons and UNC-17 (LX929) is expressed in over 120 cholinergic neurons. None of the substances led to death of neurons either after 24 h or 72 h of incubation in one of the strains.

**Figure 2:**
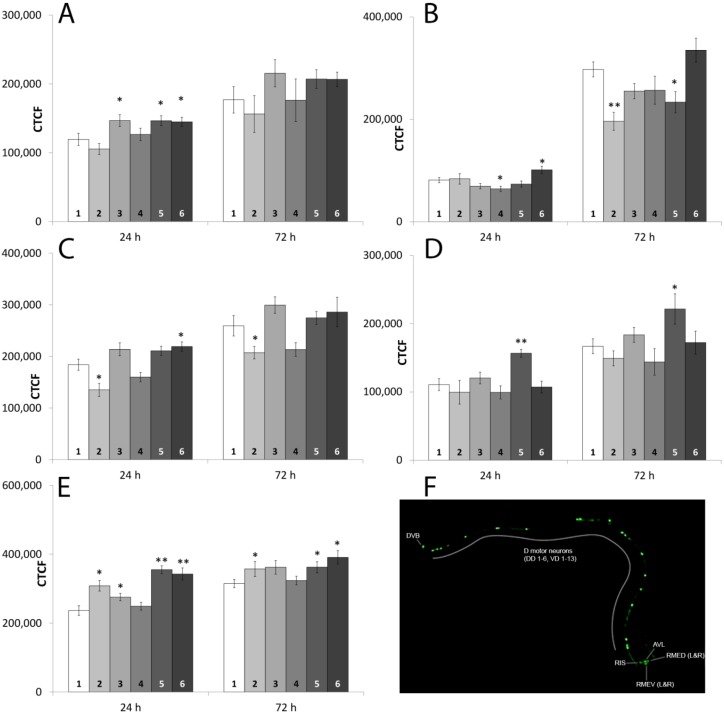
Corrected total cell fluorescence (CTCF) for the different GFP marked *C. elegans* strains after exposure to the different substances. **1**: Control, **2**: 0.1 µM TBBP-A, **3**: 50 µM TBBP-A, **4**: 50 µM DBAA, **5**: 0.1 µM TBBP-A + 50 µM DBAA, **6**: 50 µM TBBP-A + 50 µM DBAA, (**A**) strain BZ555; (**B**) strain DA1240; (**C**) strain EG1285; (**D**) strain GR1366; (**E**) strain LX929; (**F**) picture of strain EG1285 with labeled neurons as an example. Significances to the control are given by * (*p* < 0.05) and ** (*p* < 0.001). Bars represent mean values ± SEM (One Way ANOVA (Holm-Sidak-method)).

As shown in Figure 2C the amount of UNC-47 was decreased after exposure to 0.1 TBBP-A, but increased after 24 h incubation with the mixture of 50 µM TBBP-A and 50 µM DBAA. However, the quantity of protein after incubation with the mixture containing 0.1 µM TBBP-A increased in relation to its single components (Table 5), while the other mixture increased it relative to 50 µM DBAA. High concentrated TBBP-A and both mixtures increased the CTCF in BZ555 after 24 h (Figure 2A). TBBP-A (0.1 µM) and 50 µM DBAA in a mixture also increased the CTCF in relation to 0.1 µM TBBP-A. After 24 h the fluorescence of strain DA1240 was decreased by 50 µM DBAA. The mixture of the high organobromine concentrations increased the CTCF in relation to the control and to 50 µM DBAA (Figure 2B). However, after 72 h the amount of EAT-4 decreased in all exposures containing 0.1 µM TBBP-A. After 24 h, all substances except 50 µM DBAA increased the amount of UNC-17 in strain LX929 (Figure 2E). Both mixtures also increased the fluorescence in relation to at least one of their individual components. An increased expression was still detectable after 72 h of incubation with 0.1 µM TBBP-A and both mixtures. As seen in Figure 2D, expression of THP-1, marked in strain GR1366, was increased by the mixture of 0.1 µM TBBP-A and DBAA in relation to the control and to both compounds after 24 and 72 h. Table S7 displays the mean values, standard error of the mean and significances.

**Table 5:** Significances between the mixtures and single compounds.

Strain	*t*_ex_	0.1 T + 50 D Relative to		50 T + 50 D Relative to	
		0.1 T	50 D	50 T	50 D
**BZ555**	24 h	*	-	-	-
	72 h	-	-	-	-
**DA1240**	24 h	-	-	*	**
	72 h	-	-	*	*
**EG1285**	24 h	**	**	-	**
	72 h	**	*	-	*
**GR1366**	24 h	*	**	-	-
	72 h	*	*	-	-
**LX929**	24 h	*	**	-	**
	72 h	-	-	-	*

***t*_ex_**: exposure time; **0.1 T**: 0.1 µM TBBP-A; **50 T**: 50 µM TBBP-A; **50 D**: 50 µM DBAA; **red**: significant higher than the single substance; Significant changes are given by * (*p* < 0.05) and ** (*p* < 0.001).

### 2.2. Chronic Exposure

Impact of chronical exposure on neuronal development was determined by exposing eggs to the organobromines. For the tests L1-, L2/L3-, L4- and A1-developmental stages were used.

#### 2.2.1. Neuromolecular Experiments

**Figure 3:**
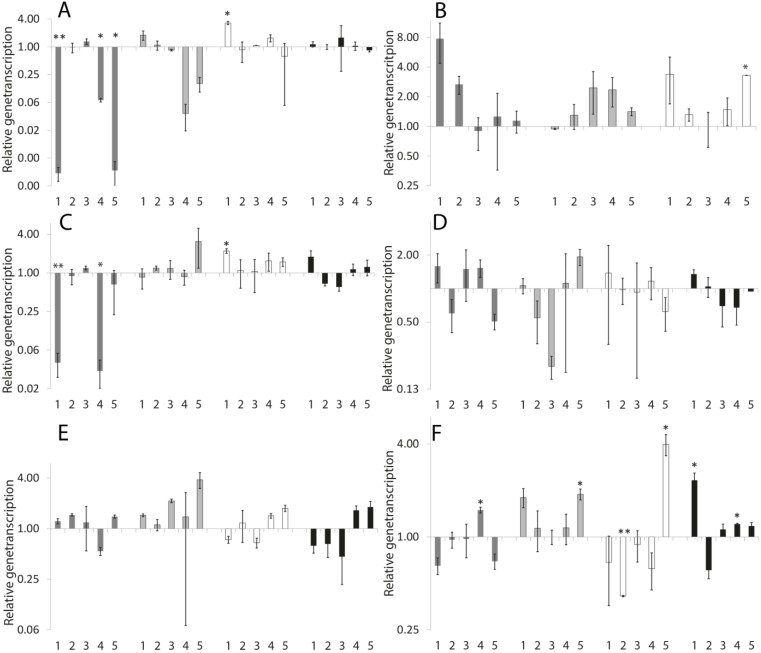
Relative gene transcription after exposure to the substances during different developmental stages. Statistical significances to the control are shown as * (*p* < 0.05) and ** (*p* < 0.001). Bars represent means ± SEM (One Way ANOVA (Holm-Sidak-method)). **Dark grey bars**: L1-stage, **light grey bars**: L2/L3-stage, **white bars**: L4-stage, **black bars**: A1-stage, **1**: 0.1 µM TBBP-A, **2**: 50 µM TBBP-A, **3**: 50 µM DBAA, **4**: 0.1 µM TBBP-A and 50 µM DBAA, **5**: 50 µM TBBP-A and 50 µM DBAA, (**A**) *casy-1*; (**B**) *dat-1*; (**C**) *eat-4*; (**D**) *tph-1*; (**E**) *unc-17*; (**F**) *unc-47*.

As shown in Figure 3A, transcription of *casy-1* was delayed by 0.1 µM TBBP-A and by both mixtures. However, 0.1 µM TBBP-A caused an increased transcription in the L4-stage. Similarly, all testing approaches containing 0.1 µM TBBP-A led to a significant decreased *eat-4* transcription in L1-stage (Figure 3C). An induction was detected in stage L4 after incubation with 0.1 µM TBBP-A. An increased transcription was also observed for *unc-17* after incubation with 50 µM DBAA and with the high concentrated mixture at L2/L3-stage (Figure 3E). However, DBAA caused a decrease in stage A1. The selected tolerance level of 2 was exceeded; also no significant changes to the control could be determined. In stage L2/L3, 50 µM DBAA caused a reduction of the transcription of *thp-1* to factor 0.2 (Figure 3D), but, due to high deviations, there was no statistical difference to the control. As shown in Figure 3B, 0.1 µM TBBP-A and 50 µM TBBP-A caused a more than two-fold induction of *dat-1* in L1-stage, and there was an induction by 50 µM DBAA and the mixture containing 0.1 µM TBBP-A in stage L2/L3. The stimulating effect of 0.1 µM could also be seen in L4-stage. However, again due to high deviations of the single data, no statistical significance could be derived. The mixture containing the high concentrations of organobromines led to a significantly increased transcription in L4-stage. A1-stage has to be excluded because all repeats for the control-group led to no results. The mixture of 0.1 µM TBBP-A and DBAA caused a significant increased transcription of *unc-47* in L1-stage and A1, even if the tolerance level of 2 was not exceeded (Figure 3F). In addition 0.1 µM TBBP-A caused a significant increased transcription in stage A1. The mixture of 50 µM TBBP-A and DBAA significantly increased the amount of mRNA in L2/L3- and L4-nematodes, while 50 µM TBBP-A repressed transcription in L4- and A1-stage. For detailed values please refer to Table S8.

#### 2.2.2. Fluorescence-Microscopic Observations of GFP Fusion-Proteins

**Figure 4:**
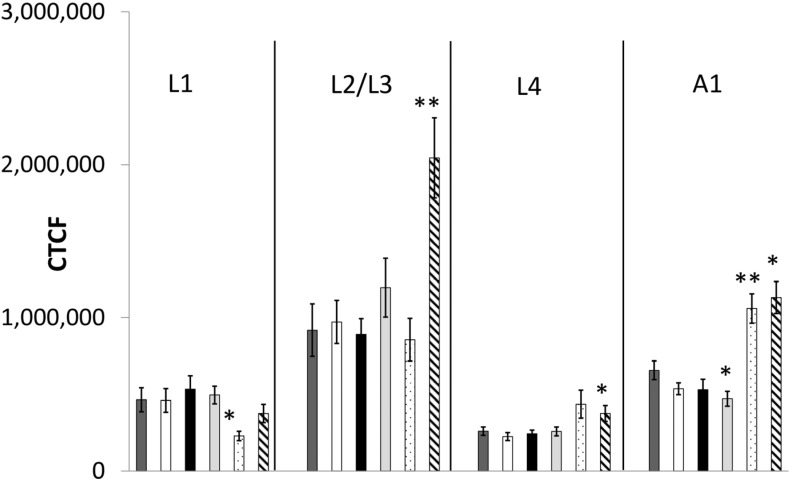
Corrected total cell fluorescence (CTCF) of *C. elegans* strain LX929 after exposure to the different substances during different developmental stages. Bars represent means ± SEM (One Way ANOVA (Holm-Sidak-method)). **Dark grey bars**: Control, **white bars**: 0.1 µM TBBP-A, **black bars**: 50 µM TBBP-A, **light grey bars**: 50 µM DBAA, **spotted bars**: 0.1 µM TBBP-A + 50 µM DBAA, **stripped bars**: 50 µM TBBP-A + 50 µM DBAA. Significances are shown as *p* < 0.05 (*) and *p* < 0.001 (**).

The influence of the substances on protein quantity and neuronal development was only examined by using *C. elegans* strain LX929, because the early development stages of all other GFP strains had insufficient fluorescence intensity. With exception of stage L1, incubation with the mixture of the high concentrated organobromines led to significant increased fluorescence intensity (Figure 4 and Table S9). The mixture containing 0.1 µM TBBP-A provoked a delayed expression of UNC-17. L1-stage showed a massive decrease of intensity, while in stage L2/L3 a trend towards an increase was observed. In stage A1 the expression was significantly increased in relation to the control, however the expression of UNC-17 was significantly decreased after exposure with 50 µM TBBP-A.

## 3. Discussion

### 3.1. Increased Pumping Frequency Leads to Increased Growth

**Figure 5:**
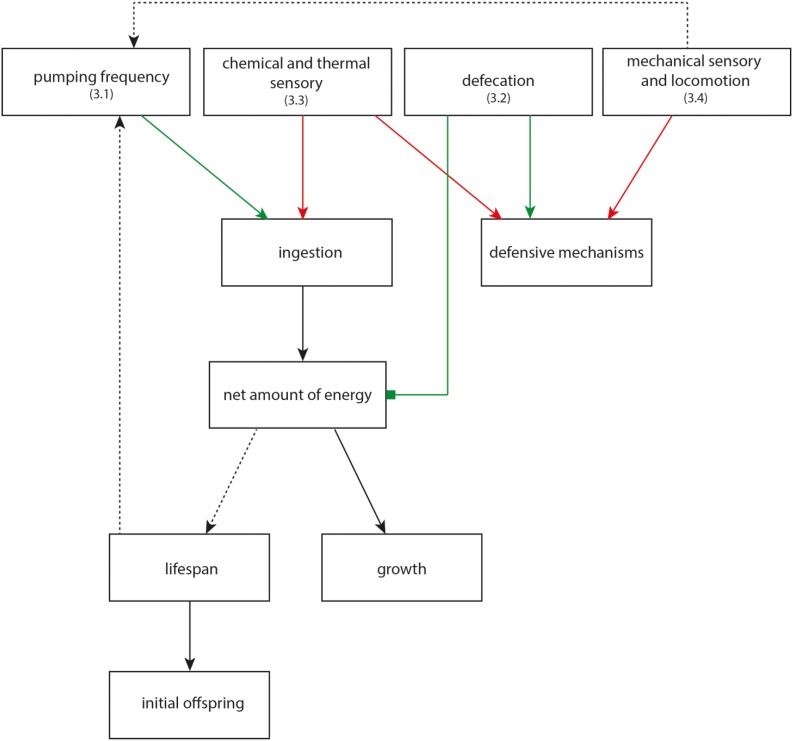
Linking between the analyzed physiological and neuronal behaviors. Identification beneath the neurophysiological parameters (**3.x**) refers to the subchapter, where this connection is discussed. **Dotted lines**: possible connection, **solid line**: proved connection, ▬►: reverse effect, ▬■: inverse effect, **red**: influence may appear under natural conditions, **green**: influence under laboratory conditions.

An increased pumping frequency leads to a faster intake of food overall. In addition, the distribution of ingested food is supported [20], thus nutrients could be absorbed easier [21]. The organism could therefore rely on better energy support which could be distributed between growth and maintenance [22]. Figure 5 gives an overview of possible associations between pumping and growth and lifespan, respectively. Green connections thereby represent influences that have been observed under laboratory conditions, whereas red connections represent influences that may increase the toxicity under natural conditions (such as decreased detection of food and stressors by decreased chemical perception) but should not do so under artificial conditions and feeding *ad libitum*.

In the present study, an increased pumping frequency was found after incubation with 0.1 µM TBBP-A, 50 µM DBAA and the mixture of both. This mainly correlates with the increase in growth previously determined by Saul *et al.* [7]. Because of the eutelie in *C. elegans* [23], growth cannot be achieved by an increased cell-number. Thus the increase in body length was caused by increased cell-size [24] which is caused, among other things, by increased storage of transcription- and translation-products [25,26]. This hypothesis is supported by the fact that 50 µM DBAA increases the biosynthesis [7]. The increase of body length therefore is most likely caused by the intensified uptake of food.

In the present study the increased pumping frequency mainly correlates with the prior detected delayed senescence, an effect that has been reported before by Wilson *et al.* [27] and Liu *et al.* [28]. Because of the stimulating effect of 50 µM DBAA on biosynthesis and metabolism [7], an energy-saving mode, as required for calorie restriction [29,30], is probably not the reason for the life-extension. According to the life-history theory [31], the increased amount of energy by enhanced pumping could not only be used for growth but also for maintenance. Thus, damage occurring for example on the DNA can be repaired much more efficiently, which may lead to a delayed senescence.

The inverse conclusion is also possible: the increased pumping frequency does not cause the life-extension but is caused by it. Pumping frequency decreases with increasing age and therefore is a measure of individual fitness [32]. Substances delaying senescence also cause individuals to be “younger” after the same time has passed. Thus these nematodes would have higher pumping frequencies than untreated worms.

**Figure 6:**
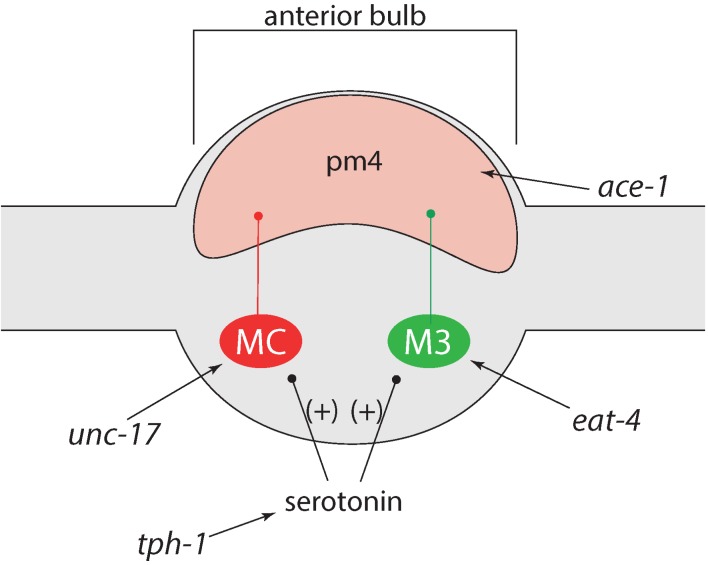
Regulation of pumping-mechanism, graph based on [36]; **MC** (**red**) and **M3** (**green**) are pharyngeal motor neurons, **pm4** are muscle cells in the metacorpus.

Pumping frequency is modulated by the MC (L&R) and M3 (L&R) neurons [33,34], which use acetylcholine and glutamate, respectively [35] (Figure 6). Transcription of *eat-4*, a glutamate transporter, and *unc-17*, an acetylcholine transporter, were significantly increased after incubation with the mixture of 50 µM of both organobromines. Both are vesicular transporters that reflect the amount of transmitter stored in the presynaptic neuron. The increased amount of both neurotransmitters might be the reason for the increased pumping frequency. However, pumping was also increased after exposure to 0.1 µM TBBP-A, 50 µM DBAA and the mixture of both, while transcription of both genes was not stimulated. Therefore, we did not get a unitary picture about how pumping is modulated by the organobromines.

### 3.2. Reduced Defecation Interval Correlates with Short Lifespan

With a shorter defecation interval the food spends less time in the intestine and the nutrients can be taken up less effectively. The resulting energy deficit may lead, as explained in Section 3.1, to a reduced body size and lifespan (Figure 5). With exception of the low concentrated TBBP-A, all substances caused a significant decrease after 24 h of incubation. After 72 h, all toxic chemicals still reduced the interval, while the mixture of the stimulating substances provoked an increase. A decreased interval may be the reason for the examined life-reduction after incubation with 50 µM TBBP-A and the mixture of the high concentrated organobromines [7].

The faster defecation after 24 h could be a protective reaction against potential toxic chemical stressors, as many studies show interconnections between enhanced defecation and stress [37,38]. This assumption is supported by the fact, that after 72 h, all neuro-stimulating substances no longer decrease the time between two defecations, but the neuro-toxic substances still do so; 0.1 µM TBBP-A could be too low concentrated to initiate the protective reaction.

**Figure 7:**
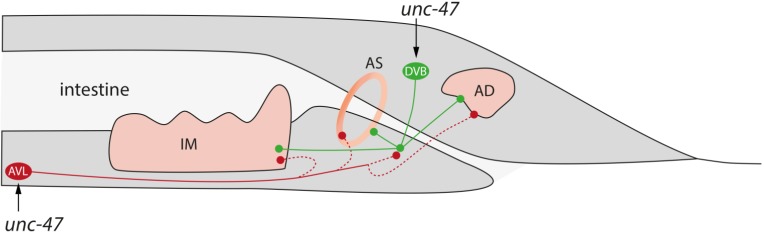
Regulation of the expulsion step of the defecation cycle, created on information from [41] and [42]; **IM**: intestinal muscle, **AS**: anal sphinter, **AD**: anal depressor, **DVB** (**green**) and **AVL** (**red**): polymodale interneurons/motor neurons; It is unclear, whether AVL acts directly on the muscles or via DVB, therefore dotted lines are used to show the connections.

Muscle contraction (intestinal muscles, anal-sphinkter and anal-depressor) on the expulsion step is mediated through the GABAergic neurons AVL and DVB [39,40] (Figure 7). Expression of the vesicular GABA transporter UNC-47, which is required to load GABA into synaptic vesicles in the presynaptic neuron, was increased after incubation with 50 µM TBBP-A and both mixtures. The resulting faster muscle contraction speed may be the reason for the shortening of the defecation interval. As the molecular methods are much more sensitive than the phenotypical observations, the minor repression of *unc-47* by 50 µM DBAA may indicate the counter-reaction to the prior stress-induced reaction. This is supported by the fact that after 72 h phenotypical an increased defecation was no longer detectable.

### 3.3. Reduced Chemical and Thermal Perception Reduces Net-Energy

The main reason for ingestion is to maximize the net-energy-uptake, the difference between energy gained from food and energy upset to receive it. [43]. The chemo-sensorial system of *C. elegans* is quite firmly set and is used for detection and evaluation of food sources [44,45]. Reduced chemical perception thereby may negatively affect the energy balance (Figure 5) and may also decrease the ability to avoid infective bacteria and fungi [46].

The high concentrated TBBP-A and the mixture of the 50 µM organobromines decreased the chemical perception. This may cause a decreased detection and evaluation of food, resulting in a lower amount of energy by the consumed food. The energy upset to receive the food would rise at the same time, because fast and targeted searching is affected. As explained in Section 3.1, this loss of energy can cause decreased body length, reproduction, and perhaps faster senescence. *In natura* with limited food available, the toxic effect of the substances could be enhanced by this feature. Under the artificial circumstances in the laboratory and feeding *ad libitum*, however, reduced chemo-perception most likely does not display a negative effect and can probably be ruled out as reason for the observed reduction of lifespan and offspring [7].

Thermal perception allows *C. elegans* to seek out the temperature where enzymes have optimal functionality. Disturbance of the thermo-sensory system can lead to developmental delays and sterility [47] and therefore may also affect lifespan [48]. All substances had only a poor impairment on isothermal behavior. As a consequence, no indication on disrupted thermotaxis as a reason for shortened lifespan could be found.

**Figure 8:**
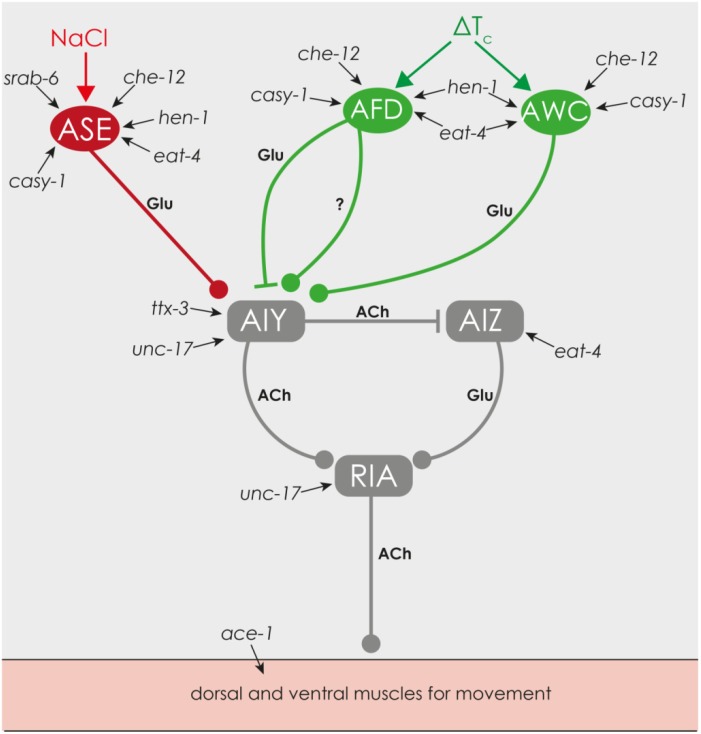
Regulation of chemical and thermal sensory, created on information from Biron *et al.* [56], Kimata *et al.* [57] and [49]. **ASE**, **AFD** and **AWC**: sensorial neurons, **AIY**, **RIA** and **AIZ**: interneurons, **red**: chemical sensory, **green**: thermal sensory, **Glu**: glutamate, **ACh**: acetylcholine, **?**: unknown mechanism, ▬●: excitatory, ▬▌: inhibitory, **Δ*T*_c_**: deviation from cultivating temperature *T*_c_.

Perception of sodium and chloride and thermal perception is mediated by the glutamatergic neurons ASE, ASH, and AWC [49,50] (Figure 8). Transcription of *hen-1*, which is needed for different types of learning, including salt- and thermotaxis [51], was decreased by the combination of the high concentrated substances and may be the reason for the reduced chemotaxis. Neither *eat-4* [52] and *casy-1* [53], nor *che-12* [54] or *srab-6* [55] seem to influence chemical and thermal perception. For detailed information about the gene function please refer to Table 2.

### 3.4. Reduction of Mechanical Sensory and Locomotive Behavior Decrease Chances of Survival in Nature

Besides chemosensory, *C. elegans* detects food sources also by mechanical stimuli and decreases its movement speed and increases pumping after detection [58,59]. A disturbed mechanical sensory thus leads to decreased intake (Figure 5) and may result, as explained in Section 3.1, in reduced body size and lifespan. A mixture of highly concentrated organobromines caused decreased mechanical sensory, lifespan and offspring. Contrary to expectations, pumping frequency was significantly increased (see Section 2.1.2). Therefore reduced food uptake and thus decreased energy cannot be the reason for the decreased lifespan. Perception of chemical and osmotic stress is mediated by the same neurons that also detect mechanical stress; and *C. elegans* reacts on immediate change of moving direction [60,61]. Disturbed perception, as caused by the mixture of both high concentrations, therefore may cause a decreased detection of harmful substances and animal and fungal predators [62,63]. Under natural conditions this may affect vitality functions in a negative way, but under controlled laboratory circumstances it can be excluded as reason for shorter lifespan. However, the lack of significant influence with the other test approaches may indicate pleiotropic toxicity such as chemical habituation or even neurodegeneration. Future studies, using strains, where all glutamatergic neurons are tagged, to evaluate the impact of the substances to neuronal survival, could help to gain a deeper insight into the processes.

After 24 h, only 50 µM DBAA impacted the relative move length. After 72 h, the speed was significantly decreased by 50 µM TBBP-A and by both mixtures. These substances also caused a decreased lifespan [7]. In the protected laboratory environment with feeding *ad libitum*, food limitation, due to reduced mobility, can be excluded as a reason for the shorter lifespan, but may apply *in natura* and therefore amplify the toxicity of a substance. After 24 h, 0.1 µM TBBP-A, 50 µM DBAA and mixture of both significantly increased the number of body bends, after longer exposure the mixture caused a decrease. An increased number of body bends may allow a faster reaction to mechanical, chemical and thermal stressors and therefore increase the survival in nature. Under laboratory conditions, this issue should not have any influence.

**Figure 9:**
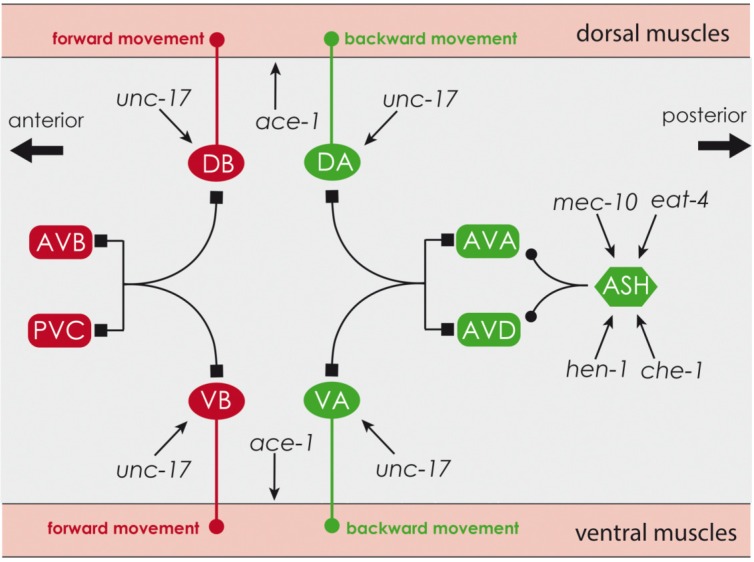
Overview of neurons and genes involved in movement and mechanotaxis, created on information from [70], Riddle *et al.* [71] and [50]; **DB**, **DA**, **VB**, **VA**: motor neurons, **AVB**, **PVC**, **AVA**, **AVD**: interneurons, **ASH**: sensory neuron, ▬●: chemical synapses, ▬■: electrical synapses, **red**: forward movement, **green**: backward movement.

Mechanical and chemical stimuli are perceived by the ASH neurons [64] and information is forwarded via Glutamate as shown in Figure 9. The mixture of both 50 µM organobromines significantly decreased transcription of the sodium channel *mec-10* and of *hen-1*, involved in the integration of sensory stimuli. Both could be a reason for the phenotypical detected reduction of mechanical sensitivity. Neither the glutamate transporter *eat-4* nor *che-12* [54,64] seem to have an influence.

Reduced reaction could be caused by habituation [65], as impairment of neuronal signal transmission by glutamate and dopamine leads to faster short-time habituation [66]. None of the genes involves in glutamate- and dopamine-pathway, respectively were decreased by the mixture of the high concentrated organobromines. Therefore habituation can be excluded as a reason for decreased mechano-sensitivity. Transcription and translation of *unc-17*, a vesicular acetylcholine transporter, involved in presynaptic storage of acetylcholine, were significantly increased by 0.1 µM TBBP-A and by the mixture containing 0.1 µM TBBP-A. This may lead to an increased number of body bends, since information is forwarded to the muscles via acetylcholine (Figure 9) [67,68]. The amount of UNC-17 was also increased by 50 µM TBBP-A and by the mixture of the high concentrations, but only in combination with an increased transcription of the acetylcholinesterase ACE-1. Increased supply by UNC-17 and degradation of acetylcholine by ACE-1 may balance each other out and, therefore, no change in the number of body bends may be observed. Relaxation is mediated via GABA distribution [69], however a connection between modified transcription of the GABA receptor *ggb-1* and the GABA transporter *unc-47* and changes in body bend frequency could not be determined.

### 3.5. Neuro-Ontogenesis

In nature, xenobiotics rarely occur for a short time interval and animals often do not have the opportunity to evade. They are therefore exposed for a long period of time and mostly in different developmental stages [72]. In addition to acute exposure on organobromines after short-term exposure (24 h and 72 h on L4- to A3-stages), gene-transcription in different stages of development and after different times of exposure have been determined to get hints of the chronical effect of the substances. Large fluctuations in transcription were observed. L1-stages often showed strong induction or repression of genes, decreasing with increasing age and exposure-period. Simultaneously, strong oscillations were observed between the different stages.

Induction of stress related genes occurs immediately after appearance of the stressor and is typically time-limited and followed by counter-regulations and negative feedback-loops [73]. Most likely, this holds also true for the examined genes, as all are involved in neuronal information forwarding. Wu *et al.* [74] obtained similar results when determining the stress response to aluminum nano-particles. Sahara *et al.* [75] showed, that yeast cells respond to cold temperatures with oscillating gene transcription and translation. The reason could be the high energy consumed on maintaining the initial stress response over a long time [76]. The reaction to chemical stress also is obviously stage-specific. Therefore a breadboard construction determining gene transcription in different stages but after identical exposure times could give interesting results.

Overall, the results of our ontogenetic experiment showed that both, exposure time and developmental stage have a great influence on the molecular reactions. Therefore these factors must be critically looked at when comparing qRT-PCRs or genome-wide DNA microarrays.

## 4. Material and Methods

### 4.1. Strains

*C. elegans* strains LX929 (vsIs48[unc-17::GFP]), DA1240 (adls1240[lin-15(+)eat-4::sGFP]X), EG1285 (oxls12[unc-47::GFP+lin-15(+)]), GR1366 (mgls42[tph-1::GFP+pRF4(rol-6(su1006))]) and BZ555 (egls1[dat-1p::GFP]) were used for the fluorescence microscope observations. Details on the function of marked genes can be found in Table 2. The remaining tests were performed using the wild-type *C. elegans* strain N2 (Bristol). All strains were obtained from the Caenorhabditis Genetics Center (CGC) (Minneapolis, MN, USA). Nematodes were maintained on 96 mm nematode growth medium (NGM) plates seeded with *Escherichia coli* strain OP50 at 20 °C [77]. The starting exposure stages were L4 larvae for acute exposure and egg stage for chronically exposure.

### 4.2. Organobromines and Exposure

The organobromines TBBP-A (TCI Europe N.V., Belgian; purity >97%) and DBAA (Fluka Analytical, Sigma-Aldrich, St. Louis, MO, USA; purity >98%) were added in concentrations of 0.1 µM and 50 µM TBBP-A, or 50 µM DBAA to the NGM agar and bacteria. DBAA (0.1 µM) was not tested, because it showed hardly any effect in the previously performed bioassay and microarray study [7]. In addition to the single substances, mixtures containing 0.1 µM TBBP-A and 50 µM DBAA and 50 µM of both substances were tested. Dimethyl sulfoxide (final concentration of 0.3% [v/v]) was used in all experiments as solvent and control.

Acute exposure included incubation for 24 h and 72 h at 20 °C to prior synchronised L4 larvae according to Ju *et al.* [6]. Chronically exposure included incubation of eggs until grown to desired examination stage (L1, L2/L3, L4 or A1). Prior to all experiments nematodes were synchronized [77,78].

### 4.3. Neurophysiologic Experiments

Synchronised L4 larvae were exposed for 24 h and 72 h, respectively at 20 °C to the organobromines. Methods used in this study followed the previously developed protocols for neurotoxic evaluation to *C. elegans* [6] and are briefly described below. Each assay was repeated independently at least three times and, unless otherwise stated, 10 nematodes per treatment and assay were used. An earlier, less comprehensive set of the data of the 50 µM concentrations of both compounds has been published before [79]. For sake of comparison, we included these data in the graphs and maintained the same conditions and units for the neurobehavior experiments.

The complete data set serves as a basis for the biomolecular and the chronic experiments, as well as for the comparison between the effect of single substances and mixtures.

#### 4.3.1. Autonomic Behavior

Body bend frequency and relative move length were determined to analyze the locomotive behavior [80]. A single body bend was determined as two complete changes of direction of the anterior part of the nematode during the sinusoidal movement. Movelength was ascertained by measuring the crawler lanes produced after transfer to fresh plates for 20 s using a VHX-600 digital microscope (Keyence, Osaka, Japan) and Image-Pro Plus software. The mean body size of every group was used for normalization. Pharyngeal pumping was quantified using a VHX-600 digital microscope by counting up and down movement of the grinder over 60 s. Defecation was assayed as described by Hart [80]. The interval between two posterior body-wall contractions was measured using a stereo microscope.

#### 4.3.2. Mechanical Sensory Stimulus

Mechanical sensory perception was determined on bacteria-free plates using the nose touch assay previously described by Liedtke *et al.* [81] and a stereo microscope. The anterior part of a forward moving nematode was touched with a fine hair ten times, interrupted by short periods of resting. Percentage of positive responses was calculated.

#### 4.3.3. Chemical Sensory

The assay for chemical sensory was conducted following Ward [82]. A 96 mm bacteria-lacking assay plate (5 mM potassium phosphate, pH 6.0, 1 mM CaCl_2_, 1 mM MgSO_4_, 20 g/L agar) was prepared and stetted: The starting point (“S”), equidistant (3 cm) to this a NaCl-spot (“N”) and a control-spot (“C”). A sodium chloride gradient was established for 24 h at “N” using an agar plug excised from a NaCl plate (prepared as above but with the addition of 100 mM NaCl). Immediately prior to the assay the agar plug was removed and 1 µL 0.5 µM sodium azide was spotted on both, “N” and “C”. Thirty previously exposed worms per plate were transferred to “S” and incubated for 1 h at 20 °C. The number of animals around “N” and “C” was determined using a stereo microscope. The chemical index (*C*_i_) was calculated using Equation (1), with *n*_N_ as number of animals counted within an area of 1.5 cm of the center of “N”and *n*_C_ number of animals counted around “C”.

(1) $$C_{\text{i}}=\frac{n_{\text{N}}-n_{\text{C}}}{30}$$

The assay was repeated four times and because of large deviations between the controls the values were normalized.

### 4.4. Neuromolecular Experiments

In order to verify neuromolecular changes after acute exposure to the substances, untreated, synchronized L4 larvae were transferred to the exposure plates and incubated for another 24 h at 20 °C. The young adults were harvested by rinsing off with cold M9 buffer, washed several times, shock frozen in liquid nitrogen and stored at −80 °C. Per treatment five repeats with two 96 mm plates, each containing about 8000 worms, were carried out.

To ascertain changes after chronical exposure, synchronized eggs were transferred directly to plates containing the test substances. After grown to the desired stage at 20 °C the nematodes were harvested as described above. Due to less organic material, four 96 mm plates with about 8700 nematodes were used for stages L1 and L2/L3 and two plates for stages L4 and A1. For each condition, samples were cultivated in triplicate.

#### 4.4.1. Selected Genes

Altogether 17 genes were selected to be examined via qRT-PCR for transcriptional changes after exposure to the substances. Genes where selected after analysis of a microarray [7] or because of possible involvement in the examined neurobehavior. Table 2 gives an overview on the function of the genes.

#### 4.4.2. RNA Preparation

RNA was isolated using the innuSPEED Tissue RNA Kit (Analytik Jena, Jena, Germany), but using lysis tube C instead of lysis tube P, and samples were homogenized with a SpeedMill Plus (Analytik Jena, Jena, Germany). The RNA was eluted using 50 µL RNase-free water. Quality and quantity of RNA was analyzed by gel electrophoresis and a spectrophotometer (NanoDrop 2000, Thermo Scientific, Waltham, MA, USA) and stored at −80 °C.

#### 4.4.3. qRT-PCR

To denaturize the secondary structure 0.8 µL oligodT were added to 10 µL RNA and incubated at 70 °C for 5 min. After chilling for a few moments 3.0 µL 5× RT buffer, 0.8 µL 10 mM dNTPs and 0.4 µL M-MLV (Moloney Murine Leukemia Virus) reverse transcriptase (Promega, Madison, WI, USA) were added and incubated for 90 min at 42 °C. The synthesized cDNA was diluted 1:20 using 5 mM TRIS/HCl buffer pH 8.0.

Primers for the genes (Table S6) were designed using Clone Manager Suite 7, manufactured by Invitrogen and set to 50 nmol/L with bidest. water. Efficiency was determined using a series of dilution (1:10; 1:50; 1:250 and 1:500) and BIORAD MyiQ™ iCyclers (BIORAD, Munich, Germany) and the iQ™5 Optical System Software.

Quantitative Real-Time Polymerase Chain Reaction (qRT-PCR) was performed with the BIORAD MyiQ iCycler and the qRT-PCR Green Core Kit (Jena Bioscience, Jena, Germany). Annealing temperatures were adjusted recoding to supplement Table S6. The quality of qRT-PCR products was determined via gel electrophoresis.

All expression levels were normalized to qRT-PCR values of reference genes *act-1* and *cdc-42*. Up- and down-regulation in relation to control was defined as a fold change by a factor of at least 1.5 for acute exposure and, due to less repeats, of 2 for chronical exposure.

### 4.5. Specific Gene Expression in Neurons

To evaluate neuronal changes five *C. elegans* stains were chosen with different types of neurons all tagged with GFP. Exposed nematodes were anaesthetized with 25 mM tetramizol and stimulated by λ_475_ using an Eclipse E200 fluorescence microscope. The signal of the whole animal was recorded with a digital microscope (digital amplification: 6 dB, edge enhancements: +4.1, exposure time: BZ555: 1 s, LX929: 0.2 s, DA1240: 1.5 s; EG1285: 1 s and GR1366: 2 s) intensity was measured using Image J software and the CTCF (corrected total cell fluorescence) was calculated with CTCF = Integrated Density − (Area of selected cell × Mean fluorescence of background). For acute exposure a 10x objective was used for all pictures. For chronical exposure only strain LX929 provided a sufficient signal to detect the early development stages. Stages L1 to L4 were recorded using a 20× objective, A1 stages with a 10× lens. The exposure times were adjusted as follows: L1: 1 s, L2/L2: 0.5 s, L4: 0.2 s and A1: 0.2 s. In order to examine the effect of organobromines after acute exposure on neuronal survival, the number of neurons after exposure was counted out for the two strains BZ555 and EG1285. At least 15–20 worms per treatment were evaluated for every experiment.

### 4.6. Statistical Significance

All statistical calculations were performed using SigmaStat 3.5 software and One Way ANOVA test (Holm-Sidak-method) and all data are displayed as mean ± SEM (standard error of the mean). Changes were considered statistically significant if their *p*-value was less than 0.05 (*) and less than 0.001 (**).

## 5. Concluding Remarks

During its life, *C. elegans* faces many challenges in its natural habitat that require behavioral adaptations. Disorders of neuronal behavior and restrictions in the initiated reactions via electrical and chemical cascades therefore represent a high risk for individuals and populations. In the present study, the nematodes were exposed to a distinct stressor to evaluate the reaction and the results showed great impact on neuronal behavior. In nature most organisms live close to their tolerance limit and therefore appear to be more vulnerable to additional occurring stresses as caused by xenobiotics than under laboratory conditions [83]. Substances that did not cause a reaction in the present study, may even affect the neurophysiology in a negative way under natural conditions. Transcription and behavior of *C. elegans* underlie a circadian rhythm [84] and are also modulated by light and temperature [85]. This means that, although our results reflect only one detail of the potential response of *C. elegans* to neuro-active substances, it enhances the scientific knowledge on the effects of organobromines. The hormetic effect of tetrabromobisphenol-A and the stimulating of dibromoacetic acid found by Saul *et al.* [7] could be confirmed on the neurological level. In addition, the effect on some genes, involved in the neuronal and chemical pathways that underlie autonomic and sensorial behavior were worked out. Exposure to the mixtures and ontogenetic studies give first hints on how xenobiotics affect behavior under more natural conditions. Therefore every anthropogenic substance released into an ecosystem is of potential risk and should be reduced to a minimum. This study also shows the importance of including neurological examinations into risk assessment and that *C. elegans* makes an ideal model organism for this.

## Supplementary Files

Supplementary File 1
