# Supplementary material for: Natural Marine and Synthetic Xenobiotics Get on Nematode’s Nerves: Neuro-Stimulating and Neurotoxic Findings in Caenorhabditis elegans
Source: Mar Drugs. 2015 May 6;13(5):2785–812. doi: 10.3390/md13052785 (PMC4446606; doi:10.3390/md13052785)
Supplement: Supplementary File 1 [file marinedrugs-13-02785-s001.pdf]

Supplementary Information

Table S1. Mean value (MV) and standard error of the mean (SEM) for locomotion behavior after 24 and 72 h of incubation. Additionally, significances (*p*) in relation to the control are shown.

	**Body Bends**			
	**24 h**		**72 h**	
	**MV ± SEM [min^{-1}]**	***p***	**MV ± SEM [min^{-1}]**	***p***
**Control**	62.46 ± 0.89		53.79 ± 1.05	
**0.1 µM TPBB-A**	66.7 ± 1.35	0.01	57.52 ± 0.77	0.01
**50 µM TPBB-A**	64.55 ± 1.79	0.31	52.1 ± 0.77	0.2
**50 µM DBAA**	69.4 ± 1.39	<0.001	56.19 ± 0.86	0.09
**0.1 µM TPBB-A + 50 µM DBAA**	71.78 ± 1.79	<0.001	52.22 ± 0.76	0.24
**50 µM TBBP-A + 50 µM DBAA**	62.59 ± 0.94	0.94	47.50 ± 0.98	<0.001
	**Relative Movelength in 20 s**			
	**24 h**	**72 h**	**24 h**	**72 h**
	**MV ± SEM**	***p***	**MV ± SEM**	***p***
**Control**	5.38 ± 0.17		5.18 ± 0.15	
**0.1 µM TPBB-A**	5.67 ± 0.20	0.263	5.50 ± 0.14	0.192
**50 µM TPBB-A**	5.52 ± 0.21	0.614	4.63 ± 0.13	0.002
**50 µM DBAA**	5.86 ± 0.19	0.064	5.10 ± 0.14	0.461
**0.1 µM TPBB-A + 50 µM DBAA**	5.48 ± 0.17	0.674	4.80 ± 0.16	0.035
**50 µM TBBP-A + 50 µM DBAA**	5.57 ± 0.13	0.370	4.05 ± 0.09	<0.001

Table S2. Mean value (MV), standard error of the mean (SEM) and significance (*p*) relative to the control for the pharynx-pumping after different times of incubation.

	**Pumping-Frequency**			
	**24 h**		**72 h**	
	**MV ± SEM [min^{-1}]**	***p***	**MV ± SEM [min^{-1}]**	***p***
**Control**	281.38 ± 2.87		265.85 ± 3.9	
**0.1 µM TPBB-A**	302.8 ± 5.78	0.002	295.49 ± 7.3	0.002
**50 µM TPBB-A**	276.01 ± 6.41	0.461	273.86 ± 5.96	0.316
**50 µM DBAA**	307.08 ± 3.11	<0.001	311.73 ± 4.49	<0.001
**0.1 µM TPBB-A + 50 µM DBAA**	341.88 ± 6.75	<0.001	323.48 ± 10.05	<0.001
**50 µM TBBP-A + 50 µM DBAA**	287.98 ± 2.93	<0.001	287.47 ± 3.45	<0.001

Table S3. Time between two defecations. Listed are the mean values (MV), standard error of the mean (SEM) and significances (*p*).

	**Time between Defecation**			
	**24 h**		**72 h**	
	**MV ± SEM [s]**	***p***	**MV ± SEM [s]**	***p***
**Control**	51.04 ± 0.67		70.39 ± 1.89	
**0.1 µM TPBB-A**	51.83 ± 1.16	0.560	73.74 ± 2.61	0.306
**50 µM TPBB-A**	47.73 ± 0.87	0.004	54.66 ± 2.04	<0.001
**50 µM DBAA**	45.93 ± 0.83	<0.001	67.83 ± 1.08	0.260
**0.1 µM TPBB-A + 50 µM DBAA**	48.39 ± 1.19	0.065	78.18 ± 2.60	0.019
**50 µM TBBP-A + 50 µM DBAA**	44.90 ± 0.75	<0.001	55.45 ± 1.55	<0.001

Table S4. Percentage of affirmative responses to mechanical stimuli to the anterior part of *C. elegans*. Mean values (MV), standard error of the mean (SEM) and significance (*p*) to the control are shown.

**Percentage of Affirmative Responses to Mechanical Stimuli**				
	**24 h**		**72 h**	
	**MV ± SEM [%]**	***p***	**MV ± SEM [%]**	***p***
**Control**	84.33 ± 2.61		86.33 ± 2.56	
**0.1 µM TPBB-A**	82.00 ± 2.73	0.804	87.33 ± 2.03	0.761
**50 µM TPBB-A**	82.67 ± 2.44	0.643	79.63 ± 3.35	0.114
**50 µM DBAA**	84.33 ± 2.48	1.000	83.00 ± 2.80	0.383
**0.1 µM TPBB-A + 50 µM DBAA**	85.67 ± 2.28	0.702	84.14 ± 2.70	0.557
**50 µM TBBP-A + 50 µM DBAA**	67.00 ± 4.82	0.004	73.00 ± 4.63	0.014

Table S5. The table shows the mean value (MV) of the chemical indices, as well as the standard error of the means (SEM) and significances (*p*) in relation to the control.

**Chemical Index Ci**				
	**24 h**		**72 h**	
	**MV ± SEM**	***p***	**MV ± SEM**	***p***
**Control**	0.28 ± 0.00		0.39 ± 0.00	
**0.1 µM TPBB-A**	0.27 ± 0.02	0.665	0.30 ± 0.09	0.415
**50 µM TPBB-A**	0.23 ± 0.05	0.007	0.26 ± 0.02	0.001
**50 µM DBAA**	0.28 ± 0.04	0.945	0.29 ± 0.06	0.210
**0.1 µM TPBB-A + 50 µM DBAA**	0.27 ± 0.06	0.736	0.12 ± 0.02	<0.001
**50 µM TBBP-A + 50 µM DBAA**	0.12 ± 0.07	0.035	0.22 ± 0.07	0.035

Table S6. Sequences for the primers for examined genes, fw: forward, rv: reverse; bp: base pairs; *T*m: melting temperature, *T*a: annealing temperature, R^2: coefficient of determination.

Gene	Primer	Sequence 5′ to 3′	GC [%]	Primer Length [bp]	*T*m [°C]	*T*a Single [°C]	*T*a [°C]	Primer Efficiency [%]	R^2	Product Length [bp]
act-1	act-1 fw	TCCAAGAGAGGTATCCTTAC	45	20	55.25	53.75	52.8	109.0	0.992	169
	act-1 rv	CGGTTAGCCTTTGGATTGAG	50	20	57.30	55.80				
cdc-42	cdc-42 fw	ATTACGCCGTCACAGTAATG	45	20	55.25	53.75	52.8	93.8	0.996	248
	cdc-42 rv	ATCCCTGAGATCGACTTGAG	50	20	57.30	55.80				
ace-1	ace-1 fw	TCTGCCTTCGACGATATTAG	45	20	55.25	53.75	52.8	102.8	0.981	235
	ace-1 rv	AGTGTCCTCACTCTGAATAC	45	20	55.25	53.75				
casy-1	casy-1_2 fw	CGAAGGCAGAAGAGACAGAG	55	21	60.90	59.40	58.4	99.7	0.992	297
	casy-1_2 rv	ACGAGCGTTCGTTGAGATGG	55	21	60.90	59.40				
che-12	che-12 fw	GAAGCATGCGATTTCACAAC	45	20	55.25	53.75	52.8	106.7	0.977	257
	che-12 rv	CTTATCCGCCCATTCATCAC	50	20	57.30	55.80				
dat-1	dat-1_3 fw	CGAAGAGGGAAAGAGCAATG	50	20	57.30	55.8	53.9	101.2	0.988	193
	dat-1_3 rv	TGGTCAGAAGACGGAACAG	52	19	56.41	54.91				
dop-1	dop-1_2 fw	AGAAAGCACGTCTTACATTG	40	20	53.20	51.70	50.7	114.4	0.995	263
	dop-1_2 rv	CTTATTTAGATCAGGCTCTTCC	40	22	56.15	54.65				
dop-3	dop-3_2 fw	ATTCGCATTCCTGTAAATCG	40	20	53.20	51.70	50.7	130.3	0.997	346
	dop-3_2 rv	ATTGTGAGCTTAGCAGTTTC	40	20	53.20	51.70				
eat-4	eat-4 fw	CCCACAGCAAATGCAAGAAG	50	20	57.30	55.80	54.8	104.8	0.853	311
	eat-4 rv	CCGGAATCTGAGTGACTAAG	50	20	57.30	55.80				
gbb-1	gbb-1 fw	TCACAACTCGCAGTGTCAAC	50	20	57.30	55.80	54.8	85.0	1	127
	gbb-1 rv	CGCAGCTTCAGCAATTACAG	50	20	57.30	55.80				
grd-12	grd-12 fw	CTCCGCAACAGCGATTCAAC	55	20	59.35	57.85	56.8	117.0	0.997	221
	grd-12 rv	CGGTGGAGGTCTAACGTAAG	55	20	59.35	57.85				

Table S6. *Cont.*

hda-4	hda-4 fw	TTCCAGTTGGACAGGATCTC	50	20	57.30	55.80	54.8	113.9	0.988	269
	hda-4 rv	TTCGCCTTCTGGATCTTCTC	50	20	57.30	55.80				
hen-1	hen-1 fw	GAGCATTTGGTCACGGAAATC	47	21	57.62	56.12	55.1	111.3	0.991	223
	hen-1 rv	GTTGCGAGGGTCATTTATGTC	47	21	57.62	56.12				
mec-10	mec-10 fw	ACAGAGCTGCATGGGTATTC	50	20	57.30	55.80	54.8	137.5	0.996	265
	mec-10 rv	AATGCTTCCGAGCTGCTATC	50	20	57.30	55.80				
srab-6	srab-6 fw	ATGAGGAGCTGAGGGCTAAG	55	20	59.35	57.85	56.8	127.3	0.990	195
	srab-6 rv	TGAACGCGACATAGGTGGAG	55	20	59.35	57.85				
tph-1	tph-1 fw	GGATCTGATGATGCGACAAC	50	20	57.30	55.80	54.8	117.6	0.997	97
	tph-1 rv	GCCCTGCTCCATACATAATCAC	50	22	60.25	58.75				
ttx-3	ttx-3 fw	AGCGGAAGCGTCTACCGATG	60	20	61.40	59.90	58.9	101.8	0.982	156
	ttx-3 rv	GCGGCTAGCTGCTTCAAGTC	60	20	61.40	59.90				
unc-17	unc-17_2 fw	GAAATGCGTCCTGGTGATAG	52	19	56.41	54.91	53.9	85.9	0.992	205
	unc-17_2 rv	GGGTTCCTTGAACCTTCTC	52	19	56.41	54.91				
unc-47	unc-47 fw	TTCAGGCAGCATGGAATGTCAC	50	22	60.25	58.75	57.8	96.9	0.967	336
	unc-47 rv	ATCCGGCTTTGTCAACACTTGG	50	22	60.25	58.75				

Table S7. Corrected total cell fluorescence for the different *C. elegans* strains marked with GFP. Shown are the mean values ± standard error of the mean, as well as significances with * $p < 0.05$ and ** $p < 0.001$.

EG1285	24 h	72 h
**Control**	183,839 ± 10,650	259,351 ± 19,644
**0.1 µM TBBP-A**	135,114 * ± 12,707	207,184 * ± 12,131
**50 µM TBBP-A**	213,880 ± 12,550	299,439 ± 16,138
**50 µM DBAA**	159,973 ± 9090	213,449 ± 13,160
**0.1 µM TBBP-A + 50 µM DBAA**	210,991 ± 8948	274,663 ± 12,422
**50 µM TBBP-A + 50 µM DBAA**	219,156 * ± 8983	285,905 ± 28,598
**BZ555**	**24 h**	**72 h**
**Control**	119,346 ± 8832	176,944 ± 19,205
**0.1 µM TBBP-A**	105,338 ± 8007	156,333 ± 26,699
**50 µM TBBP-A**	146,758 * ± 8579	215,485 ± 19,607
**50 µM DBAA**	126,570 ± 8940	176,236 ± 31,027
**0.1 µM TBBP-A + 50 µM DBAA**	146,494 * ± 7074	207,074 ± 13,680
**50 µM TBBP-A + 50 µM DBAA**	144,842 * ± 6608	206,656 ± 10,525
**DA1240**	**24 h**	**72 h**
**Control**	81,409 ± 4906	297,624 ± 14,638
**0.1 µM TBBP-A**	83,751 ± 10,186	196,252 ** ± 17,541
**50 µM TBBP-A**	69,415 * ± 5332	255,222 ± 14,992
**50 µM DBAA**	64,218 ± 5198	256,876 * ± 27,374
**0.1 µM TBBP-A + 50 µM DBAA**	73,585 ± 5941	233,562 ± 20,874
**50 µM TBBP-A + 50 µM DBAA**	101,324 * ± 7177	335,110 ± 23,297
**LX929**	**24 h**	**72 h**
**Control**	236,746 ± 14,116	315,214 ± 11,903
**0.1 µM TBBP-A**	308,748 * ± 15,462	357,166 * ± 21,528
**50 µM TBBP-A**	275,864 * ± 10,755	362,078 ± 19,764
**50 µM DBAA**	249,282 ± 11,207	323,964 ± 12,539
**0.1 µM TBBP-A + 50 µM DBAA**	355,086 ** ± 10,842	362,292 * ± 15,940
**50 µM TBBP-A + 50 µM DBAA**	342,870 ** ± 17,143	390,778 * ± 19,565
**GR1366**	**24 h**	**72 h**
**Control**	110,876 ± 8678	167,014 ± 10,928
**0.1 µM TBBP-A**	99,574 ± 17,346	149,276 ± 11,013
**50 µM TBBP-A**	120,510 ± 8529	183,670 ± 10,933
**50 µM DBAA**	99,341 ± 9685	143,921 ± 19,431
**0.1 µM TBBP-A + 50 µM DBAA**	156,732 ** ± 5764	221,663 * ± 22,235
**50 µM TBBP-A + 50 µM DBAA**	107,262 ± 8587	172,394 ± 16,767

Table S8. Relative gene transcription after chronic exposure. Mean value ± standard error of the mean are shown, as well as significances with p < 0.05 (*) und p < 0.001 (**). **1**: 0.1 µM TBBP-A; **2**: 50 µM TBBP-A; **3**: 50 µM DBAA; **4**: 0.1 µM TBBP-A + 50 µM DBAA; **5**: 50 µM TBBP-A + 50 µM DBAA.

**Developmental stage**	**Gene**	**casy-1**	**dat-1**	**eat-4**	**thp-1**	**unc-17**	**unc-47**
**L1-Stage**	1	0.00 ** ± 0.00	7.75 ± 3.40	0.04 ** ± 0.02	1.59 ± 0.47	1.23 ± 0.09	0.65 ± 0.08
	2	0.91 ± 0.23	2.67 ± 0.56	0.89 ± 0.24	0.60 ± 0.20	1.46 ± 0.05	0.96 ± 0.11
	3	1.31 ± 0.18	0.90 ± 0.33	1.20 ± 0.08	1.50 ± 0.73	1.19 ± 0.65	0.97 ± 0.24
	4	0.07 * ± 0.01	1.26 ± 0.90	0.03 * ± 0.01	1.53 ± 0.28	0.54 ± 0.06	1.49 * ± 0.06
	5	0.00 * ± 0.00	1.14 ± 0.29	0.66 ± 0.44	0.51 ± 0.08	1.39 ± 0.06	0.69 ± 0.08
**L2/L3-Stage**	1	1.80 ± 0.41	0.94 ± 0.01	0.86 ± 0.30	1.06 ± 0.17	1.44 ± 0.06	1.80 ± 0.26
	2	1.09 ± 0.25	1.30 ± 0.37	1.19 ± 0.09	0.55 ± 0.23	1.11 ± 0.17	1.14 ± 0.34
	3	0.85 ± 0.03	2.46 ± 1.13	1.17 ± 0.38	0.20 ± 0.05	2.14 ± 0.11	1.00 ± 0.11
	4	0.04 ± 0.02	2.35 ± 0.78	0.87 ± 0.23	1.11 ± 0.94	1.38 ± 1.31	1.15 ± 0.26
	5	0.16 ± 0.0	1.41 ± 0.13	3.07 ± 1.89	1.93 ± 0.32	3.81 ± 0.82	1.89 * ± 0.15
**L4-Stage**	1	3.29 * ± 0.26	3.37 ± 1.68	2.19 * ± 0.19	1.38 ± 1.07	0.74 ± 0.07	0.69 ± 0.33
	2	0.87 ± 0.41	1.32 ± 0.19	1.09 ± 0.51	0.98 ± 0.26	1.16 ± 0.48	0.41 ** ± 0.00
	3	1.08 ± 0.00	1.00 ± 0.39	1.05 ± 0.56	0.93 ± 0.77	0.68 ± 0.09	0.89 ± 0.21
	4	1.55 ± 0.27	1.48 ± 0.46	1.54 ± 0.48	1.17 ± 0.37	1.42 ± 0.09	0.62 ± 0.17
	5	0.62 ± 0.57	3.29 * ± 0.04	1.48 ± 0.23	0.62 ± 0.21	1.75 ± 0.15	3.98 * ± 0.62
**A1-Stage**	1	1.14 ± 0.15		1.78 ± 0.42	1.35 ± 0.13	0.63 ± 0.12	2.33 * ± 0.27
	2	1.00 ± 0.12		0.68 ± 0.06	1.04 ± 0.21	0.66 ± 0.20	0.61 ± 0.07
	3	1.59 ± 1.29		0.60 ± 0.09	0.70 ± 0.24	0.46 ± 0.25	1.12 ± 0.09
	4	1.05 ± 0.22		1.14 ± 0.23	0.67 ± 0.21	1.65 ± 0.21	1.21 * ± 0.01
	5	0.84 ± 0.06		1.24 ± 0.34	0.94 ± 0.27	1.81 ± 0.30	1.18 ± 0.06

Table S9. Corrected total cell fluorescence after chronic exposure. Mean value (MV) and standard error of the mean (SEM) are shown, as well as significances to the control with * $p < 0.05$ und ** $p < 0.001$; **c**: Control; **1**: 0.1 µM TBBP-A; **2**: 50 µM TBBP-A; **3**: 50 µM DBAA; **4**: 0.1 µM TBBP-A + 50 µM DBAA; **5**: 50 µM TBBP-A + 50 µM DBAA

	L1-Stage			L2/L3-Stage	
	**MV**	**SEM**		**MV**	**SEM**
**c**	462,578	78,058	**c**	917,722	171,419
**1**	458,555	76,725	**1**	971,382	140,736
**2**	531,912	87,496	**2**	890,164	101,965
**3**	493,932	57,382	**3**	1,195,461	192,830
**4**	228,259 *	30,333	**4**	854,931	138,951
**5**	374,019	59,387	**5**	2,043,737 **	261,800
	**L4-Stage**			**A1-Stage**	
	**MV**	**SEM**		**MV**	**SEM**
**c**	259,329	27,013	**c**	655,308	61,421
**1**	223,983	25,529	**1**	534,365	38,603
**2**	241,976	23,987	**2**	528,781	67,757
**3**	257,369	28,545	**3**	469,529 *	47,949
**4**	434,010	90,630	**4**	1,059,589 **	95,526
**5**	374,167 *	51,529	**5**	1,131,099 *	104,469

Supplements Thermotaxis

Methods

Thermotaxis Assay

Isothermal behavior was examined as described by Kuhara *et al.* [84]. 24 h prior to the experiment 96 mm bacteria-lacking assay plates were prepared using 8 mL thermo-agar (2.6 g/L NaCl [0.4 M NaCl], 25 mM potassium phosphate, 20 g/L agar). A gradient from 17 to 25 °C was established using a vial of frozen acetic acid (constant humidity of 40%–80%), placed 20 min bevor starting on the bottom of the plate. Afterwards a single worm was transferred to the 20 °C area to crawl freely for 1 h. The received trace was anayzed and categorisized. Preference to one temperature was categorisized as 17 °C, 20 °C or 25 °C, animals that moved between two areas were classified as 17 °C/20 °C and 20 °C/25 °C, respectively. The relative share for every substance and category was analyzed. The assay was repeated four times using 4–5 worms per group.

Results

Thermal sensory allows the nematode to seek out optimal temperature and decreased perception may lead to suboptimal enzyme activity. As the nematodes were fed *ad libitum* during pre-experimental cultivation, they show isothermal behavior to their breeding temperature. Therefore, when exposed to a temperature gradient, they crawl towards that temperature during the assay. A decreased isothermal behavior, seen by a more scattered distribution among the different temperatures, reflects a limited ability to percept temperature.

The control group showed a distinct isothermal behavior after both incubation periods to its cultivation temperature (20 °C). Exposure to all substances changed the behavior as shown in Figures T1 and T2 and Table T1. The nematodes were attracted to both, higher and lower temperatures; however, no statistical difference could be detected. Due to the design of the thermotaxis assay a total number of only 16 to 20 nematodes were tested per substances. To gain more significant results, the assay should be repeated in a large scale than it was possible during this study.

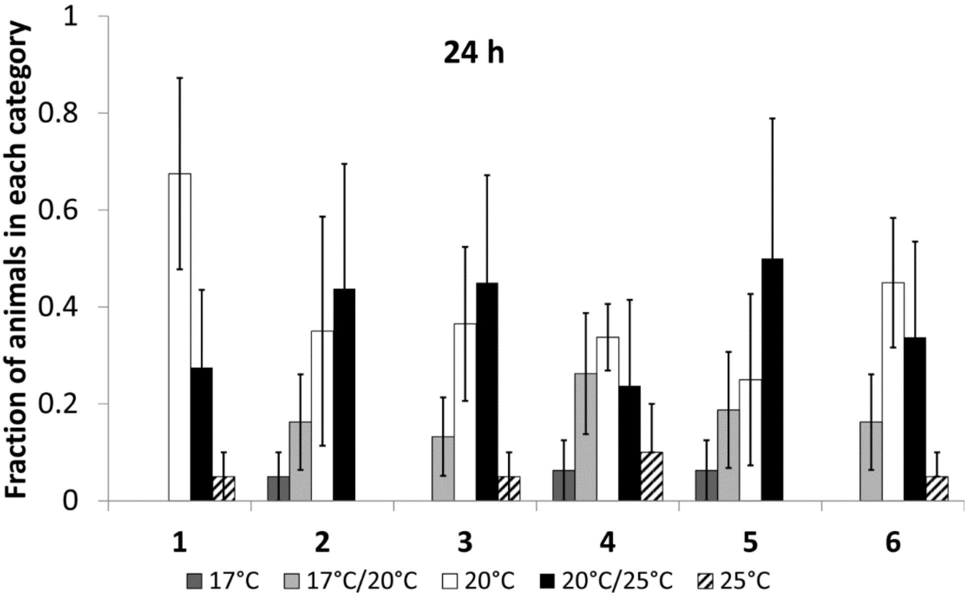

Figure T1. Thermotaxis after 24 h of incubation. **1**: Control, **2**: 0.1 µM TBBP-A, **3**: 50 µM TBBP-A, **4**: 50 µM DBAA, **5**: 0.1 µM TBBP-A + 50 µM DBAA, **6**: 50 µM TBBP-A + 50 µM DBAA. Bars represent mean value ± SEM (One way ANOVA (Holm-Sidak-method)). No value reached the significance limit of $p < 0.05$.

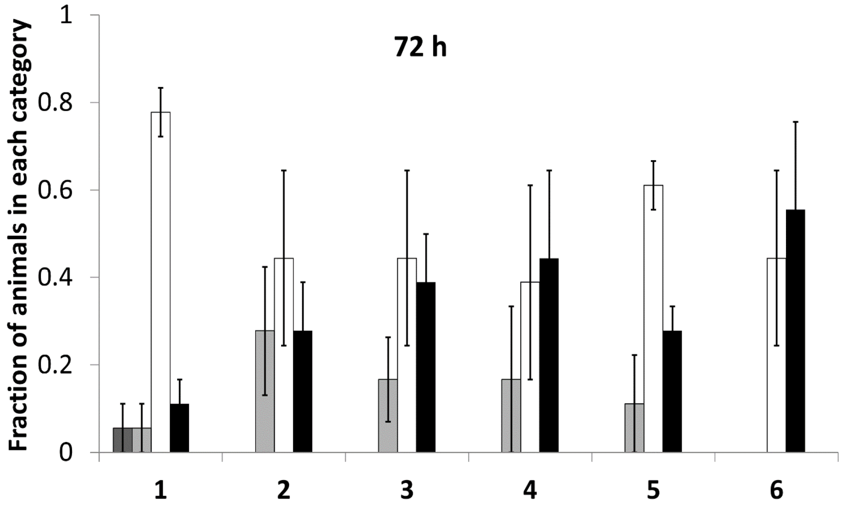

Figure T2. Thermotaxis after 72 h of incubation. **1**: Control, **2**: 0.1 µM TBBP-A, **3**: 50 µM TBBP-A, **4**: 50 µM DBAA, **5**: 0.1 µM TBBP-A + 50 µM DBAA, **6**: 50 µM TBBP-A + 50 µM DBAA. Bars represent mean value ± SEM (One way ANOVA (Holm-Sidak-method)). No value reached the significance limit of $p < 0.05$.

Table T1. The mean value (MV), standard error of the mean (SEM) and significances (*p*) are shown for the thermotaxis experiments after 24 h and 72 h of incubation.

**24 h**	**17 °C MV ± SEM (*p*)**	**17 °C/20 °C MV ± SEM (*p*)**	**20 °C MV ± SEM (*p*)**	**20 °C/25 °C MV ± SEM (*p*)**	**25 °C MV ± SEM (*p*)**
**Control**	0.00 ± 0.00	0.00 ± 0.00	0.68 ± 0.20	0.28 ± 0.16	0.05 ± 0.05
**0.1 µM TPBB-A**	0.05 ± 0.05 (0.356)	0.16 ± 0.10 (0.151)	0.35 ± 0.24 (0.332)	0.44 ± 0.26 (0.611)	0.00 ± 0.00 (0.356)
**50 µM TPBB-A**	0.00 ± 0.00 (1.000)	0.13 ± 0.08 (0.153)	0.37 ± 0.16 (0.267)	0.45 ± 0.22 (0.546)	0.05 ± 0.05 (1.000)
**50 µM DBAA**	0.06 ± 0.06 (0.356)	0.26 ± 0.12 (0.080)	0.34 ± 0.07 (0.158)	0.24 ± 0.18 (0.880)	0.10 ± 0.10 (0.670)
**0.1 µM TPBB-A + 50 µM DBAA**	0.06 ± 0.06 (0.356)	0.19 ± 0.12 (0.168)	0.25 ± 0.18 (0.160)	0.50 ± 0.29 (0.521)	0.00 ± 0.00 (0.356)
**50 µM TBBP-A + 50 µM DBAA**	0.00 ± 0.00 (1.000)	0.16 ± 0.10 (0.151)	0.45 ± 0.13 (0.382)	0.34 ± 0.20 (0.814)	0.05 ± 0.05 (1.000)
**72 h**	**17 °C MV ± SEM (*p*)**	**17 °C/20 °C MV ± SEM (*p*)**	**20 °C MV ± SEM (*p*)**	**20 °C/25 °C MV ± SEM (*p*)**	**25 °C MV ± SEM (*p*)**
**Control**	0.06 ± 0.06	0.06 ± 0.06	0.78 ± 0.06	0.11 ± 0.06	0.00 ± 0.00
**0.1 µM TPBB-A**	0.00 ± 0.00 (0.374)	0.28 ± 0.15 (0.230)	0.44 ± 0.20 (0.184)	0.28 ± 0.11 (0.251)	0.00 ± 0.00 (1.000)
**50 µM TPBB-A**	0.00 ± 0.00 (0.374)	0.17 ± 0.10 (0.374)	0.44 ± 0.20 (0.184)	0.39 ± 0.11 (0.089)	0.00 ± 0.00 (1.000)
**50 µM DBAA**	0.00 ± 0.00 (0.374)	0.17 ± 0.17 (0.561)	0.39 ± 0.22 0.165)	0.44 ± 0.20 (0.184)	0.00 ± 0.00 (1.000)
**0.1 µM TPBB-A + 50 µM DBAA**	0.00 ± 0.00 (0.374)	0.11 ± 0.11 (0.678)	0.61 ± 0.06 (0.101)	0.28 ± 0.06 (0.101)	0.00 ± 0.00 (1.000)
**50 µM TBBP-A + 50 µM DBAA**	0.00 ± 0.00 (0.374)	0.00 ± 0.00 (0.374)	0.44 ± 0.20 (0.184)	0.56 ± 0.20 (0.099)	0.00 ± 0.00 (1.000)

© 2015 by the authors; licensee MDPI, Basel, Switzerland. This article is an open access article distributed under the terms and conditions of the Creative Commons Attribution license (http://creativecommons.org/licenses/by/4.0/).
